# Molecular modelling of sorption processes of a range of diverse small organic molecules in Leonardite humic acid

**DOI:** 10.1111/ejss.12868

**Published:** 2019-08-20

**Authors:** Drazen Petrov, Daniel Tunega, Martin H. Gerzabek, Chris Oostenbrink

**Affiliations:** ^1^ Department of Material Sciences and Process Engineering, Institute of Molecular Modeling and Simulation University of Natural Resources and Life Sciences Vienna Vienna Austria; ^2^ Department of Forest and Soil Sciences Institute of Soil Research, University of Natural Resources and Life Sciences Vienna Vienna Austria; ^3^ School of Pharmaceutical Science and Technology, Tianjin University Tianjin People's Republic of China

**Keywords:** humic substance, modelling, molecular dynamics, organic matter, soil pollution, SOM, SOM dynamics, sorption, sorptivity, structure

## Abstract

Soil organic matter (SOM) is abundant in the environment and plays an important role in several biogeochemical processes, including microbial activity, soil aggregation, plant growth and carbon storage. One of its key functions is the retention and release of various chemical compounds, primarily governed by the sorption process, which strongly affects the environmental fate of nutrients and pollutants. Sorption largely depends on the composition of SOM, as well as its structure, dynamics and the thermodynamic conditions. Although several approaches are available, experimental characterization of sorption mechanisms is not easy. Computational models for predicting sorption coefficients often require a wealth of experimental data for training and are only applicable to compounds and conditions related to the training dataset. Here, we use molecular dynamics (MD) simulations to study the sorption of a range of small organic compounds. As a model SOM system we use the standard Leonardite humic acid (LHA) sample, which physicochemical properties have recently been characterized computationally in detail. This model allowed us to estimate sorption propensities of the systems at two different hydration levels (water activities close to 0 and 1), showing a remarkable correlation with experimental data. Importantly, this molecular modelling approach based on perturbation free‐energy calculations is rigorously derived from statistical thermodynamics and requires no experimental sorption data for training. It is therefore in principle applicable to any SOM model or thermodynamic condition. Moreover, the power of MD simulations to provide high‐resolution insight into atomistic and molecular interactions was employed to explore how sorbate molecules associate with the LHA matrix and which contacts they form. The heteroatoms of both sorbate and sorbent play an important role and water molecules are identified as further key players in facilitating the sorption process.

**Highlights:**

- Modelling of the sorption processes in soil organic matter at atomistic level.
- Rigorous, physics‐based approach applicable to a range of SOM systems and conditions.
- Remarkable level of matching with experimental data with additional insight into the molecular mechanism.
- Interactions between the sorbate and local environment strongly affects the sorption process.

## 1 INTRODUCTION

Soil organic matter (SOM) is a key part of the composition of soil, playing an essential role in a range of biogeochemical and environmental processes (Brady & Weil, 2008; Hartemink, Gerzabek, Lal, & McSweeney, 2014; Murphy, 2014; Stevenson, 1994). The composition of SOM varies from region to region and is dependent heavily on the utilization of the soil as well as on the vegetation cover, climate and age of the soil layer (Bayer, 2002; Campbell et al., 2000; Collins, Rasmussen, & Douglas, 1992; Ding, Novak, Amarisiriwardena, Hunt, & Xing, 2002; Havlin, Kissel, Maddux, Claassen, & Long, 1990; Nardi, Morari, Berti, Tosoni, & Giardini, 2004; Olk, 2006; Tatzber et al., 2008, 2009). The sorption and desorption of compounds such as nutrients and pollutants are some of the most important functions of SOM, which influences their transport and bioavailability significantly.

Different experimental approaches have been established to determine the strength and mechanism of sorption in SOM samples; however, this is often tedious and time consuming (Borisover & Graber, 2004; Bronner & Goss, 2011; Canan Cabbar, 1999; Dorris & Gray, 1980; Graber, Tsechansky, & Borisover, 2007; Niederer, Goss, & Schwarzenbach, 2006b). Models of experimentally determined partition coefficients between SOM and air have been combined with water/air partition coefficients to obtain SOM/water partition coefficients. Efforts to predict the partition coefficients from alternative coefficients (e.g., the octanol/water partition coefficient) or sorbate‐specific descriptors have also been described (Niederer, Goss, & Schwarzenbach, 2006a; Nguyen, Goss, & Ball, 2005; Sabljic, 1984). However, these approaches require a wealth of training data for calibration and are generally only applicable to chemically similar compounds.

The sorption process might be affected by a number of factors in addition to chemical composition of SOM, which in turn will strongly affect the environmental fate of the sorbates. For example, the structure and dynamics of SOM itself largely depend on its chemical composition, whereas varying water content causes conformational rearrangements as well as changes in flexibility and mobility of SOM molecules (Schaumann & Bertmer, 2008). Soil organic matter‐associated water also plays a significant role in enhancing or weakening the sorptive potential. Interestingly, sorption of small compounds influences SOM–water interactions as well, thus affecting its hydration state (Borisover, 2013; Borisover, Sela, & Chefetz, 2011.

To take these additional factors into account computationally, a structural modelling of SOM systems has to be considered. Modelled systems may be described at different levels of detail. For instance, quantum mechanical (QM) calculations explicitly include electron structure and provide the highest level of detail. However, QM methods are computationally rather expensive and, therefore, limited in the number of atoms and tractable timescales. On the other hand, classical modelling methods, including molecular dynamics (MD) simulations, approximate the interactions with a force field (i.e., a set of potential energy terms). Associated empirical parameters are typically derived by fitting atomic or molecular properties of small molecules against calculated quantum‐mechanical or experimentally measured data. So far, different approaches have been employed to model SOM systems (Tunega, Gerzabek, Haberhauer, Lischka, & Aquino, 2016), including several proposed model compounds (Albers, Banta, Jacobsen, & Hansen, 2008; Davies, Ghabbour, Khairy, & Ibrahim, 1997; Diallo et al., 2003; Hayes, 1989; Sein, Varnum, & Jansen, 1999), with a single structure representing the ensemble. Both quantum‐mechanical and classical methods (Aquino et al., 2009, 2011; Kubicki, 2016; Schulten & Schnitzer, 1993, 1995, 1997; Sein, Varnum, & Jansen, 1999) have been used to study proposed models, revealing possible three‐dimensional structures. Based on such techniques, modelling of sorption processes in model SOM‐molecules has been applied (Ahmed, et al. 2015; Gros, Ahmed, Kühn, & Leinweber, 2017; Kubicki & Apitz, 1999; Niederer & Goss, 2007; Schulten, Thomsen, & Carlsen, 2001; Wu et al. 2015). However, such models have mostly described SOM as a single molecule, often in a vacuum environment (possibly embedded in a polarizable continuum) or with few water molecules explicitly attached, which arguably falls short in capturing several relevant features of realistic, hydrated, multimolecular structures of SOM systems.

**Figure 1:**
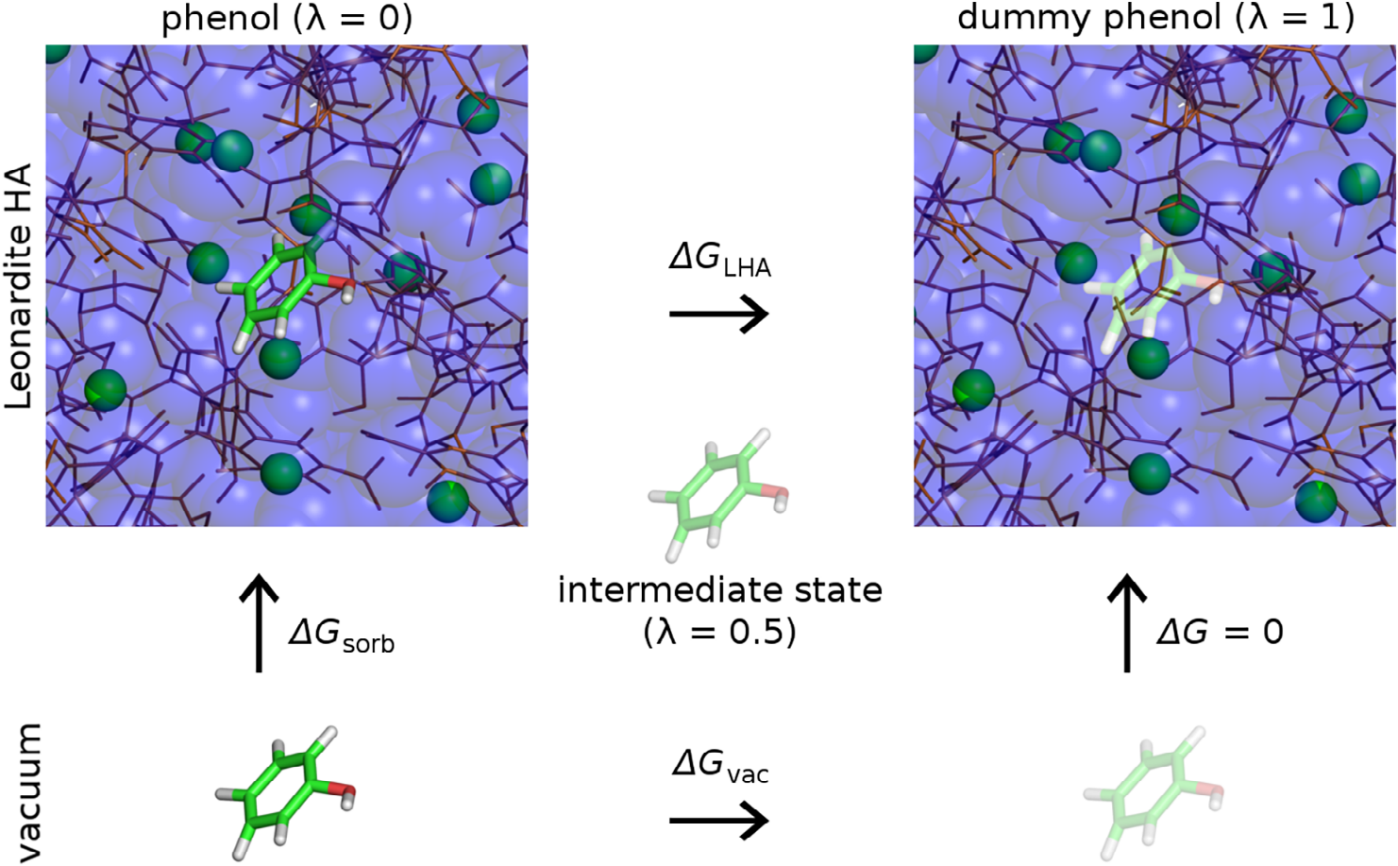
Sorption free energy of phenol in the Leonardite humic acid (LHA) model expressed as the difference in the solvation free energies in the LHA and vacuum (*ΔG*_sorb_ = *ΔG*_vac_ ‐ *ΔG*_LHA_) according to the thermodynamic cycle, as the sum of the free energy changes along the cycle is zero. Representative model of the LHA (top) with embedded phenol in its completely interacting form (*λ* = 0, left) and non‐interacting, dummy form (*λ* = 0, right). Horizontal arrows represent perturbation processes, for which the free energy changes are calculated. Water is shown in blue transparent spheres, LHA molecules in brown and line representation, and Ca ions as green spheres. Phenol is shown in stick representation, with the transparency depicting the level of perturbation

Notably, in recent years we have developed computational tools based on condensed‐phase models and classical molecular dynamics simulations that are in principle able to tackle the problem of describing SOM systems as a complex supramolecular mixture of various molecular species and to explore a range of conditions in which sorption occurs (Petrov et al., 2017; Sündermann et al., 2015). Furthermore, MD simulations allow us to calculate solvation free energy that involves decoupling a compound of interest from its surrounding environment. In such an approach, the interactions between a compound and its surrounding are scaled down to zero in a stepwise manner. The change in the free energy upon such a perturbation process, coupled to a parameter *λ*, is readily computed using thermodynamic integration or non‐equilibrium free‐energy calculations (Jarzynski, 1997; Kirkwood, 1935). The free energy of sorption from water or vacuum is directly related to the partition coefficients *K*_*SOM/water*_ and *K*_*SOM/vacuum*_, respectively, and can be expressed as the difference between the solvation free energy in water or vacuum and the solvation free energy in SOM, according to a thermodynamic cycle (Figure 1). Importantly, such a perturbation approach based on MD simulations presents a rigorous, physics‐based method able to predict the changes of the free energy in principle at the level of force field accuracy (Bruckner & Boresch, 2011; de Ruiter et al., 2013; Oostenbrink & van Gunsteren, 2006; Shirts, Mobley, & Chodera, 2007). Moreover, this approach provides a microscopic‐level insight into atomistic interactions and motions of studied processes that helps us deepen our understanding of underlying molecular mechanisms.

Here, we employ MD simulations to study sorption processes in standard Leonardite humic acid (LHA), a sample readily available from the International Humic Substances Society. It is one of the richest known sources of humic acids and its isolation, preparation and chemical composition have been documented in detail (Thorn et al., 1989) and a wealth of experimental sorption data for this material are available (Niederer et al., 2006b). We have recently generated several condensed phase models of LHA and characterized their structure, dynamics and properties in detail, giving a good agreement with experimental data (Petrov et al., 2017). In addition, we estimate the sorption propensity of a range of small organic molecules and compare the results with available experimental data. In addition, we study interactions between sorbate molecules and their surroundings, their localization in the sorbent matrix, and ask how this relates to their sorption propensities.

## 2 METHODS

### 2.1 MD simulations

Three independent equilibrated systems of the standard LHA at low water activity close to 0 (dry system with 0.2 M fraction of water) and at water activity equal to 1 (hydrated system with 0.3 M fraction of water) were taken from Petrov et al. (2017). Briefly, the models were created using the Vienna Soil‐Organic‐Matter Modeler (Sündermann et al., 2015) by generating a diverse set of relatively small molecules (40 for each of the three independent systems; i.e., 120 in total), which together form a condensed‐phase model of the LHA with corresponding chemical composition. To ensure overall neutrality, an appropriate amount of counterions was added to the systems, where Ca^2+^ ions were used for their significant role in the structure and stability of SOM (Kalinichev et al., 2011; Kloster et al., 2013). The hydration level of the LHA systems with a given water content was determined by estimating the water activity (*a*_w_) from the free‐energy difference of transferring a water molecule from bulk water to the system of interest (*ΔG*_water → LHA_) (Bakarić et al., 2018; Petrov et al., 2017), where the water activity was calculated as $a_{w}=e^{\frac{{\Delta G}_{\text{water}\to \mathrm{LHA}}}{\mathrm{RT}}}$, with *R* representing the gas constant and *T* the absolute temperature.

All molecular dynamics simulations were performed using the GROMOS11 molecular simulation package (Schmid et al., 2012) with 1 fs integration step. The united‐atom GROMOS force field, parameter set to 53A6 (Oostenbrink et al., 2004), was used to describe the LHA models as well as sorbate molecules (molecular topology files are provided as a part of SI). Water molecules were described by the SPC model (Berendsen et al., 1981). The temperature and the pressure were kept constant at 300 K and 1 bar using a weak coupling with a relaxation time of 0.1 ps and 1.5 ps, respectively (Berendsen et al., 1984). Pressure scaling was applied anisotropically, with an estimated isothermal compressibility of 3.44 × 10^−4^ (kJ mol^−1^ nm^−3^)^−1^. A molecular pair‐list was generated using a triple‐range cut‐off (Tironi et al., 1995). Non‐bonded interactions up to a short range of 0.8 nm were calculated at every time step from a pair‐list that was updated every five steps. Interactions up to a long‐range cut‐off of 1.4 nm were calculated at pair‐list updates and kept constant in between. A reaction‐field contribution was added to the electrostatic interactions and forces to account for a homogeneous medium with a dielectric permittivity of 61 outside the cut‐off sphere. The SHAKE algorithm was used to constrain the bond lengths to their optimal values with a relative geometric accuracy of 10^−4^ (Ryckaert et al., 1977).

### 2.2 Free energy calculations

Non‐equilibrium free energy calculations were carried out to compute the sorption free energies for a set of 18 small organic molecules (Table 1). The simulation conditions were the same as described above. An immersed sorbate molecule was decoupled from its surrounding by using a coupling parameter *λ*, such that at *λ* = 0, the normal Hamiltonian (*H*) is obtained, whereas at *λ* = 1, the interactions between the sorbate and its surroundings have been switched off. A soft‐core potential was used for perturbations of non‐bonded interactions (Beutler et al., 1994). To estimate the free energy change of the decoupling process, non‐equilibrium simulation was used together with the Crooks Gaussian intersection method (Goette & Grubmüller, 2009) and the Jarzynski equality (Jarzynski, 1997), where the non‐equilibrium work distribution was obtained from:

(1) $$W=\int_{0}^{1}\frac{\partial H\left(\lambda \right)}{\partial \lambda }\mathrm{d\lambda}.$$

In particular, both removing (*λ* = 0 → *λ* = 1) and growing (*λ* = 1 → *λ* = 0) a sorbate molecule within 1.6 ns simulation was repeated 25 times for three independent models for a total of 150 perturbation simulations and 240 ns per sorbate. Equilibrated systems were used as initial configurations with a sorbate molecule placed and oriented randomly within the simulation box. To obtain statistically robust results, we have resampled the non‐equilibrium work distributions 1,000 times. Reported free‐energy differences and their error estimates were calculated using the Crooks Gaussian intersection method as the averages and the standard deviations over the resampled distributions of free energies. The free energy of sorption was calculated as:

(2) $$\Delta G_{\text{sorb}}=\Delta G_{\mathrm{vac}}-\Delta G_{\mathrm{LHA}},$$

where *ΔG*_LHA_ is the free energy of decoupling of the sorbate in the standard Leonardite HA and *ΔG*_vac_ is the free energy of decoupling in a vacuum. Importantly, this property directly relates to the logarithm of the experimentally measured humic acid/air partition coefficients *K*_*iHA,air*_ (up to an elusive constant c) (Niederer et al., 2006b), as $\Delta G_{\text{sorb}}^{\exp}=-\mathrm{RT}\ln K_{\mathrm{iHA},\mathrm{air}}+c$. Furthermore, hydration free energies (i.e., the free energy of transferring a compound from vacuum to water) of the studied set of compounds, together with two additional groups of compounds chemically similar to dimethyl succinate and 1‐undecene (Table 1), were calculated using the extended thermodynamic integration (de Ruiter & Oostenbrink, 2016) approach, by carrying out five independent 200‐ps‐long equilibrium simulations at 21 equally distant *λ* points. The free energy of this process (*ΔG*_water_) is computed by integrating the ensemble average of ∂*H/*∂*λ* over *λ*, which was numerically calculated using trapezoidal integration. The hydration free energy (*ΔG*_hyd_) is then obtained as:

(3) $$\Delta G_{\mathrm{hyd}}=\Delta G_{\mathrm{vac}}-\Delta G_{\text{water}}.$$

**Table 1:** Set of diverse small organic compounds spanning a range of experimentally determined sorption coefficients, used for modelling in the context of sorption in Leonardite humic acid (LHA). Calculated sorption free energies in the dry and hydrated LHA given with the error estimate from bootstrapping in kJ Mol^−1^. Relative sorption free energies were calculated as the difference between values obtained for the hydrated and dry systems and error estimates using error propagation. Experimental sorption data for the dry and hydrated LHA taken from Niederer et al. (2006b), with the free energy of sorption scaled such that all values in the experimental set are less and equal to 0 kJ Mol^−1^

#	Compound	$\Delta G_{\text{sorb}\;\left(\mathrm{dry}\right)}^{\text{calc}}$	$\Delta G_{\text{sorb}\;\left(\mathrm{hyd}\right)}^{\text{calc}}$	$\Delta \Delta G_{\text{sorb}}^{\text{calc}}$	$\Delta \Delta G_{\text{sorb}\;\left(\mathrm{dry}\right)}^{\exp}$	$\Delta \Delta G_{\text{sorb}\;\left(\mathrm{hyd}\right)}^{\exp}$
1	Propan‐2‐ol	−36.8 ± 0.5	−15.2 ± 1.1	21.6 ± 1.2	−5.2	−9.0
2	3‐methylbutan‐1‐ol	−45.0 ± 1.8	−18.1 ± 1.1	26.9 ± 2.1	−11.6	−11.3
3	2‐methylpropan‐1‐ol	−41.6 ± 0.8	−16.7 ± 1.9	24.9 ± 2.1	−7.6	−6.5
4	Propan‐1‐ol	−41.4 ± 1.1	−23.5 ± 1.1	17.9 ± 1.6	−8.3	−9.1
5	Pentan‐1‐ol	−46.5 ± 1.1	−20.3 ± 2.4	26.2 ± 2.6	−12.4	−12.7
6	Heptan‐1‐ol	−50.4 ± 1.4	−22.2 ± 1.3	28.2 ± 1.9	−17.8	−16.8
7	Phenol	−45.8 ± 3.4	−30.3 ± 1.1	15.5 ± 3.6	−19.8	−22.0
8	Cyclopentanol	−40.2 ± 1.7	−16.9 ± 1.4	23.3 ± 2.2	−11.3	−14.1
9	p‐xylene	−19.6 ± 2.4	3.6 ± 1.4	23.2 ± 2.8	−5.6	−3.4
10	Benzaldehyde	−38.5 ± 1.2	−21.2 ± 0.8	17.3 ± 1.4	−12.0	−14.8
11	Acetophenone	−39.2 ± 2.4	−17.1 ± 1.5	22.1 ± 2.8	−17.9	−17.7
12	2‐propanone	−24.2 ± 1.2	−9.9 ± 1.0	14.3 ± 1.6	−5.2	−4.5
13	4‐methylpentan‐2‐one	−32.2 ± 0.8	−5.5 ± 1.2	26.7 ± 1.4	−7.8	−5.5
14	2‐hexanone	−34.8 ± 1.1	−8.4 ± 1.0	26.4 ± 1.5	−9.9	−7.3
15	2,4‐pentanedione	−41.1 ± 2.0	−20.0 ± 1.2	21.1 ± 2.3	−10.2	−11.6
16	Isopropylether	−29.9 ± 1.6	−0.1 ± 1.2	29.8 ± 2.0	−1.8	−1.8
17	Dimethyl succinate	−63.6 ± 2.0	−39.9 ± 1.4	23.7 ± 2.4	−19.3	−19.4
18	1‐undecene	−35.1 ± 2.2	5.9 ± 1.7	41.0 ± 2.8	−11.6	−10.6
Additional 1						
1	Ethylbutanoate					
2	Ethylpropanoate					
3	Ethylethanoate					
4	Methylpropanoate					
5	Methylacetate					
Additional 2						
1	1‐butene					
2	1‐pentene					
3	1‐hexene					

### 2.3 Trajectory analysis

Coordinates of the simulations were stored to disk every 5 ps. Simulated trajectories were analyzed primarily using gromos++ analysis tools (Eichenberger et al., 2011). Potential energy, number of hydrogen bonds, solvent‐accessible surface area and the radial distribution functions were obtained directly from simulated trajectories. Preferential solvation of a species *A* around a species *B* was calculated as:

(4) $$\delta_{\mathrm{BA}}=x_{B}\frac{G_{\mathrm{AB}}-\sum x_{\alpha }G_{\mathrm{A\alpha}}}{V_{\text{corr}}+\sum x_{\alpha }G_{\mathrm{A\alpha}}},$$

where *α*, *A* and *B* identify different species, such as sorbate, LHA, water molecules and calcium atoms. *V*_*corr*_ stands for a correlation volume (sphere with a radius of 1.5 nm) and *G*_*AB*_ for Kirkwood‐Buff integrals that were calculated from the radial distribution functions with an upper boundary of 1.5 nm. *x*_*A*_ is the molar fraction of species *A* in the system, calculated as the normalized number of heavy atoms. Moreover, Kirkwood‐Buff integrals were also evaluated with shorter upper boundaries of 0.3, 0.4 and 0.5 nm to characterize the local environment surrounding sorbate molecules. Note that non‐normalized Kirkwood‐Buff integrals (multiplied with *x*_*A*_ and *x*_*B*_) are marked with a star.

A number of properties were calculated by monitoring interactions between a sorbate and its surroundings (Table S1). In particular, potential energy and its two components (electrostatic and van der Waals) and the number of hydrogen bonds were monitored between a sorbate and LHA molecules, water and calcium ions, and between LHA molecules and water, respectively. The occurrence of a hydrogen bond was determined using a geometric criterion considering the acceptor‐hydrogen distance (at most 0.25 nm) and the donor‐acceptor angle (at least 135°). The solvent‐accessible surface area (SASA) (i.e., the surface of a molecule accessible to the solvent) of the sorbate was calculated together with the carbon and heteroatom fractions. Kirkwood‐Buff integrals, which quantify the amount of one type of molecule or particle surrounded by another type, were calculated between a sorbate and constituents of the Leonardite HA models and their subparts, including carbon and heteroatoms of the sorbate on the one hand, and carbon and heteroatoms of LHA molecules, water and calcium on the other.

## 3 RESULTS

### 3.1 Sorption of small organic molecules in the dry and hydrated Leonardite HA

We have used molecular dynamics simulations to study sorption of a set of small organic compounds (Table 1), which was compiled from a set of experimentally determined sorption coefficients (Niederer et al., 2006b), such that the range of coefficients is covered uniformly and chemical heterogeneity is achieved among the compounds. The interactions of the selected compounds with the LHA models were defined by assigning force field parameters within the framework of the united‐atom GROMOS force field (Oostenbrink et al., 2004; Petrov, Margreitter, Grandits, Oostenbrink, & Zagrovic, 2013; Soares et al., 2004). As an appropriate description of interactions between components of simulated systems is essential for utilizing molecular dynamics, we validated our parameterization by calculating hydration free energies and comparing them against experimental data. We chose this validation approach because the GROMOS force field has been parameterized against experimental hydrophobic and hydrophilic solvation data (Oostenbrink et al., 2004; Petrov et al., 2013; Soares et al. 2004). Additionally, this ensures the proper distribution of functional groups between hydrophilic and hydrophobic phases, which is expected to be crucial for accessing sorption properties of small molecules in SOM.

**Figure 2:**
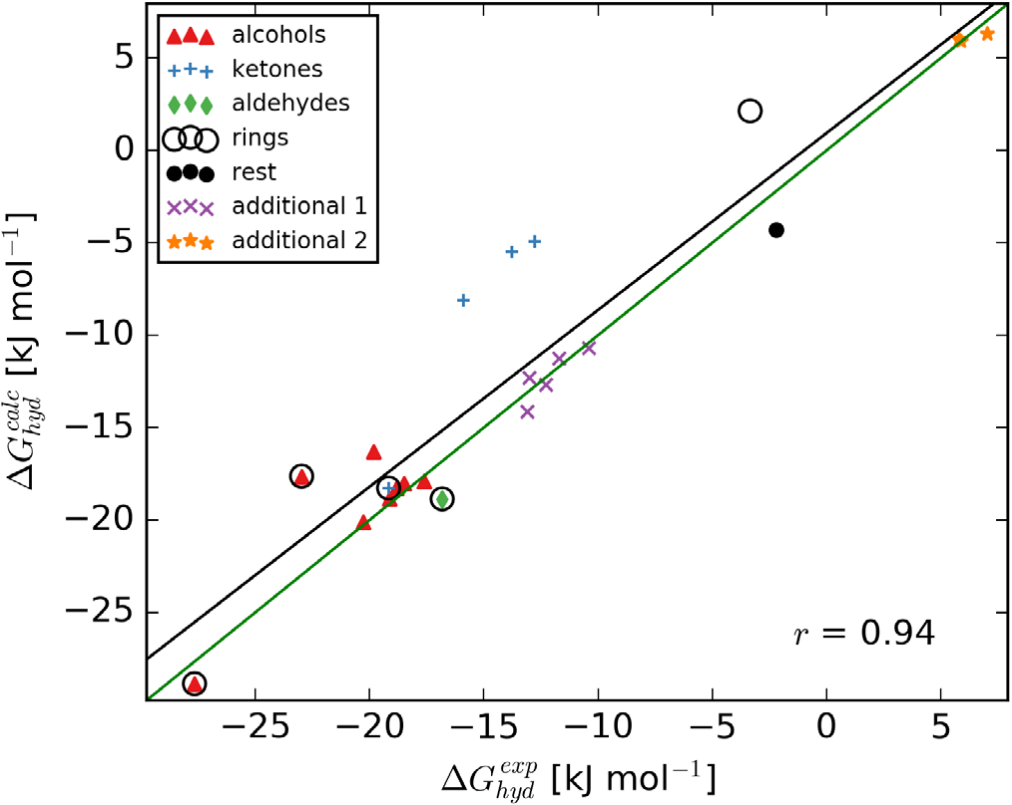
Parameterization validation through comparison between calculated and experimental hydration free energy. The correlation is captured with the black regression line and Pearson coefficient (r = 0.94), whereas the identity line is shown in green. Compounds (Table 1) are separated into groups based on their chemical composition

To the best of our knowledge, experimental hydration free energies are not available for dimethyl succinate and 1‐undecene, for which reason additional, chemically similar compounds with available experimental data were added to the set (Table 1). The initial assignment of parameters to isopropyl ether, dimethyl succinate and 1‐undecene led to discrepancies between calculated and experimental hydration free energies. To improve the matching, these three compounds were reparametrized (see molecular topology files provided as a part of Supporting Information for details), which yielded an excellent agreement with experimental data across the entire set, including the remainder of 15 compounds (Figure 2 and Figure S1), with a correlation coefficient of 0.94 and the regression line closely matching the identity line. Note that the reparameterization of dimethyl succinate, bearing two ester groups, resulted in a much stronger effect (change in hydration free energy of about 8 kJ mol^−1^) than for the corresponding group of additional compounds bearing only one.

**Figure 3:**
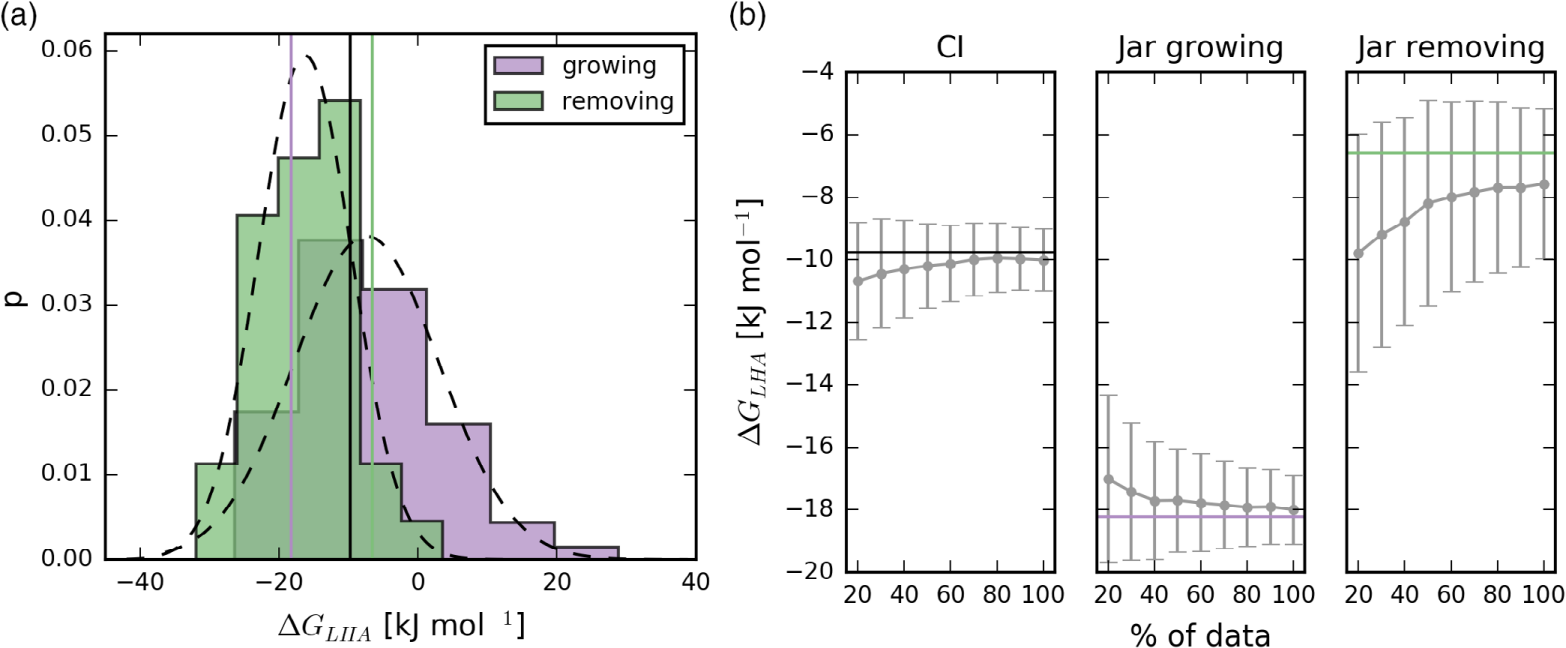
Free‐energy difference associated with the solvation of acetone in the hydrated Leonardite humic acid (LHA) models. (a) The distribution of non‐equilibrium simulations with the Gaussian fits are shown together with vertical lines representing the free energy difference calculated from full data using Crooks Gaussian intersection (CI) (black line) and Jarzynski equality (purple and green line used for growing and removing simulations, respectively). (b) Averages and standard deviations from the bootstrapping samples (1000) as error bars are shown in grey, whereas black, purple and green horizontal lines represent the free energy difference from full data

The set of compounds was subjected to calculations of sorption free energies. In particular, we performed 150 independent non‐equilibrium perturbation simulations of growing and removing a sorbate in the dry and hydrated LHA models, corresponding to the perturbation process of ‐*ΔG*_LHA_ and *ΔG*_LHA_ depicted in Figure 1, respectively. To test for potential convergence issues, we used bootstrapping to resample distributions of obtained non‐equilibrium work and calculated the sorption free energy by applying the Crooks Gaussian intersection (CI) method and Jarzynski equality a thousand times using different amounts of data. This analysis shows that the Crooks Gaussian intersection method converges faster and provides smaller uncertainty (bootstrapping estimates) than the Jarzynski approach for acetone sorbed in the hydrated LHA (Figure 3). Similar convergence behaviour was observed for other sorbates and the dry LHA as well (data not shown), and therefore we applied the CI approach to obtain the sorption free energies.

**Figure 4:**
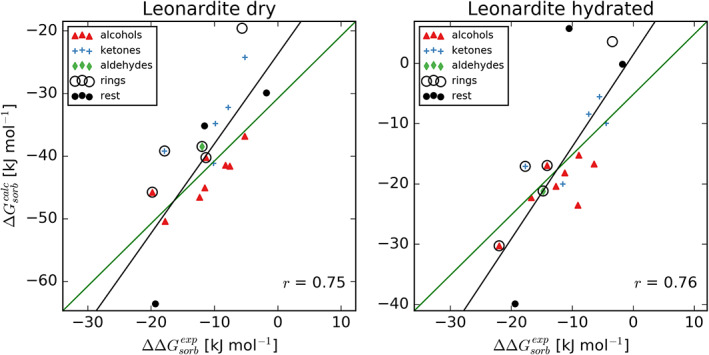
Sorption free energy in the dry and hydrated Leonardite humic acid (LHA); comparison with experimental data. The correlation is captured with the black regression line and Pearson coefficient, whereas the best fitting line with slope 1 is shown in green. Compounds (Table 1) are separated into groups based on their chemical composition

**Figure 5:**
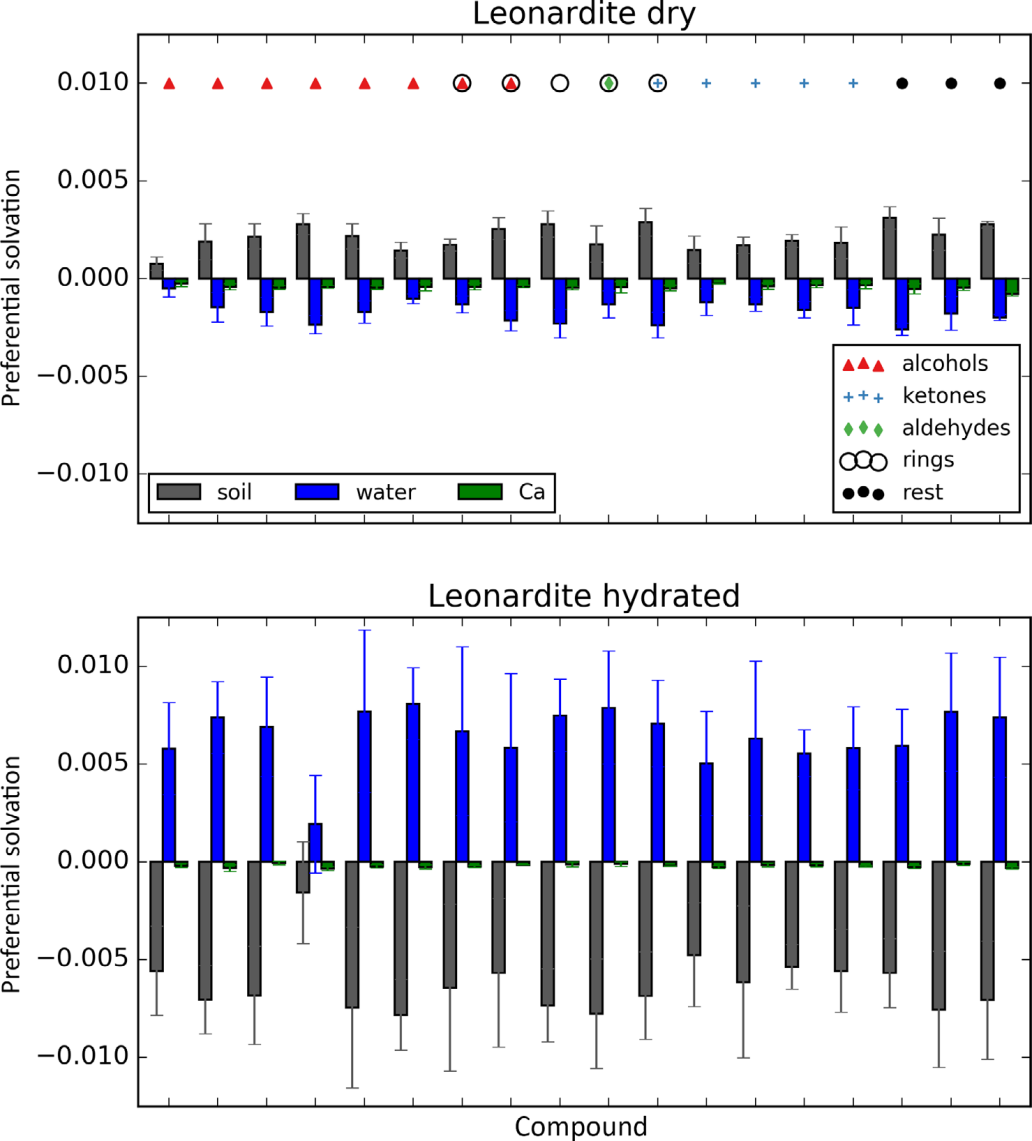
Preferential solvation of the sorbate by Leonardite humic acid (LHA) molecules (grey), water (blue) and calcium ions (green) in dry and hydrated LHA. This analysis shows that the sorbate is preferentially surrounded by the LHA molecules in the dry and by water molecules in the hydrated LHA, regardless of the sorbate molecule. Symbols at the top of the upper panel indicate the type of sorbate

The calculated sorption free energies in both dry and hydrated Leonardite HA models display a substantial level of matching to experimental data with correlation coefficients of 0.75 and 0.76, and slightly steeper regression compared to the identity lines (Figure 4). Note that due to the elusive constant c, the experimentally determined sorption free energies are only determined up to a constant shift and therefore denoted as relative values, $\Delta \Delta G_{\text{sorb}}^{\exp}$. Even with some level of disagreement, these correlations are remarkable given that the calculation approach applied is exclusively based on statistical‐mechanics principles and does not require prior calibration of the model against experimental sorption data. Several potential sources of observed discrepancies can be identified, including uncertainty in experimental measurements and calculations (estimated averages of 1.6 and 1.3 kJ mol^−1^ for the dry and hydrated LHA) or differences in modelled and experimental conditions to name a few (see Discussion for more detail). Interestingly, sorption free energies calculated in the dry Leonardite HA show a similar level of matching to experimental data measured in hydrated LHA samples (Figure S2) with a correlation coefficient of 0.77, whereas the opposite comparison yields a weaker correlation (r = 0.67). Furthermore, calculated free energies of hydration are almost identical to the sorption in hydrated LHA, to a much greater extent than experimentally observed (Figure S3). However, this water‐like behaviour in terms of sorption strength might also arise as a bias of non‐equilibrium simulations, where the sorbate localizes preferentially to water patches due to low diffusion coefficients of LHA molecules. This is further corroborated by the analysis of preferential solvation, showing that sorbate molecules indeed preferentially interact with water in hydrated LHA and with LHA molecules in the dry systems (Figure 5). On the other hand, such preferential solvation could also just be a result of a higher water content available for interactions in the hydrated system.

**Figure 6:**
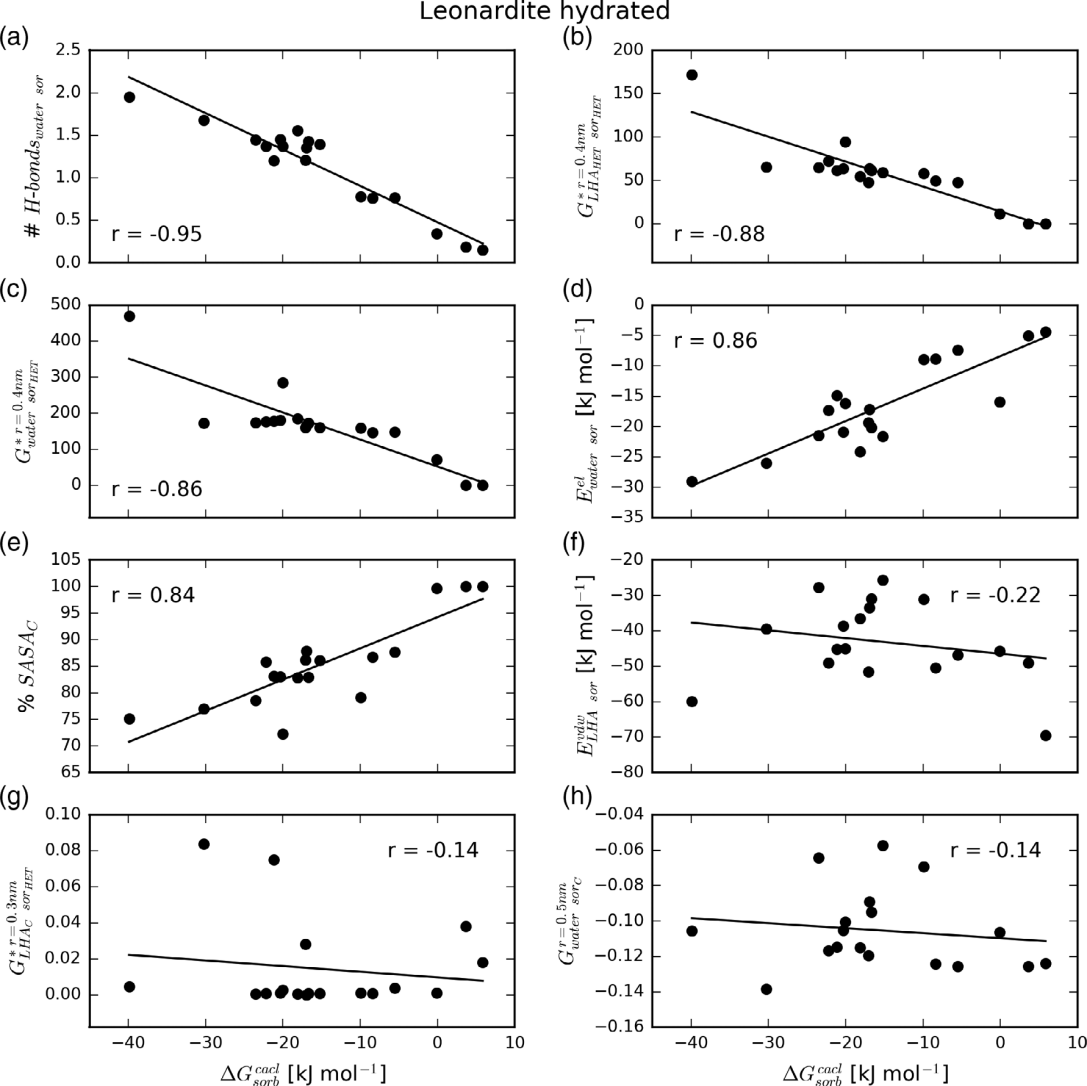
Selected microscopic properties calculated from observed interactions in simulated trajectories between sorbate compounds and Leonardite humic acid (LHA) and their relation to sorption free energy in the hydrated LHA. The correlation between a given property and the sorption free energy for the 18 studied compounds (Table 1) is captured with the black regression line and Pearson coefficient. Examples of properties showing a strong anticorrelation (a–c), a strong correlation (d) and (e), and no correlation (f–h)

**Figure 7:**
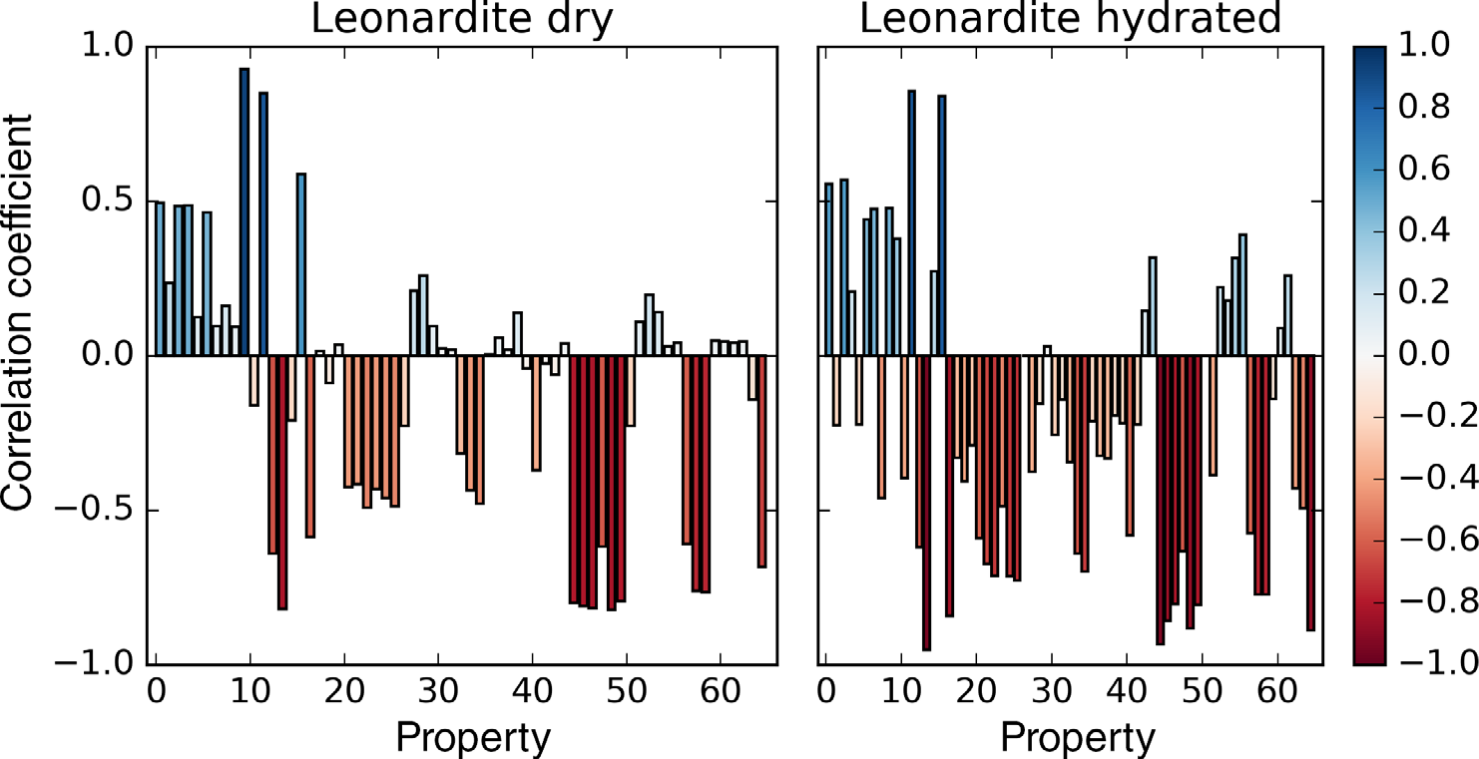
Correlations between the microscopic properties associated with interactions of a sorbate with its immediate surroundings and sorption free energy for the dry and hydrated Leonardite humic acid (LHA). A number of explored properties (Table S1), primarily related to interactions and contacts involving heteroatoms of either sorbate or LHA molecules and water molecules, exhibit a strong correlation or anticorrelation. Correlation coefficients are shown as bars coloured according to correlation strength

Importantly, this latter analysis showcases the power of the MD approach in providing high resolution insight into molecular interactions. To benefit from it further, we investigated how sorbates associate within the sorbent matrix and what type of interactions they form. In particular, we monitored a number of properties directly associated with such interactions and immediate contacts between the sorbate and its environment (Table S1), and how they relate to the sorption free energy. For example, we observed a strong anticorrelation between the sorption free energy and the number of hydrogen bonds the sorbate forms with water molecules (Figure 6a). Similarly, the number of contacts, derived from non‐normalized Kirkwood‐Buff integrals, between heteroatoms of the sorbate with water and heteroatoms of the LHA molecules (Figure 6b,c) are strongly anticorrelated, whereas the electrostatic potential energy between the sorbate and water, and the percentage of carbon in the solvent‐accessible surface area of the sorbate, are strongly correlated (Figure 6d,e) with the sorption free energy in the hydrated Leonardite HA. Similar comparisons of other explored properties (Table S1 and Figure 7) with the calculated sorption free energies revealed a high level of correlation or anticorrelation for a number of them, giving insight into the driving forces for small‐molecule sorption in LHA. The majority of these properties involve heteroatoms of the sorbate and LHA molecules, and water molecules, displaying how these types of contacts and interactions can largely affect the sorption propensity (Table S1 and Figures 6 and 7). Moreover, this highlights the importance of the local environment and the way it interacts with the sorbate. On the other hand, not surprisingly, many properties show no correlation with the sorption free energy (Figures 6f–h and 7). Importantly, a similar behaviour and relationship between sorption free energies and the evaluated properties was observed for the dry Leonardite as well (Table S1 and Figure 7 and Figure S4).

## 4 DISCUSSION

This study presents the first‐ever attempt to study sorption properties of a range of small organic molecules in standard Leonardite HA as a model SOM system, using perturbation free‐energy calculations in combination with MD simulations. In contrast to several approaches applied so far (Kubicki & Apitz, 1999; Nguyen et al., 2005; Niederer & Goss, 2007; Niederer, Goss, & Schwarzenbach, 2006a; Sabljic, 1984; Schulten, Thomsen, & Carlsen, 2001; Wu, Zhu, Ji, & Chen, 2015), this technique is solely based on statistical mechanical principles and therefore does not require calibration using experimental sorption data and is in principle able to model any type of SOM system at given thermodynamic conditions. In addition, it provides a high‐resolution insight into the molecular interactions needed to understand relevant mechanisms of the underlying processes.

Before turning our focus to sorption modelling, a quick discussion of the interaction parameters (i.e. the force field) is needed. Intrinsically, the interactions that govern atomic and molecular motions are of a quantum nature, and by using a force field they are approximated with classical potential energy terms. These approximations notwithstanding, force field methods have been successfully employed for decades to study various molecular systems (Karplus & McCammon, 2002; Orsi, 2014; van Gunsteren et al., 2006). Here, in order to be consistent with the Leonardite model created by the Vienna Soil‐Organic‐Matter Modeler, the set of studied small organic molecules was parametrized within the framework of the united‐atom GROMOS force field. Because validity of MD simulations depends strongly on the quality of force field parameters, initial parameterization of the set of sorbate molecules (i.e., assignment of force field parameters) was subjected to validation. To this end, we calculated hydration free energies and compared them to experimental data, where only three compounds (from the set of 18, Table 1, Figure 2 and Figure S1) were reparametrized to improve the match with the experimental data and obtain a satisfying set of parameters. Validation against hydration free energy was chosen because the GROMOS force field has been parameterized against experimental hydrophobic and hydrophilic solvation data (Oostenbrink et al., 2004; Petrov et al., 2013; Soares et al., 2004). These thermodynamic data are arguably one of the most important properties for ensuring the proper distribution of functional groups between hydrophilic and hydrophobic phases, which is expected to be crucial for accessing sorption properties of small molecules in soil organic matter. Note that very recently an updated version of the automated topology builder became available, leading to excellent reproduction of hydration free energies for hundreds of compounds (Stroet et al., 2018), potentially making manual reparameterization as described here obsolete.

As the diffusion of molecular species in SOM samples is significantly reduced compared to the diffusion in water (Petrov et al., 2017), the resulting free energy is expected to be strongly dependent on the initial placement of the solute. Applying equilibrium methods such as thermodynamic integration would potentially lead to biased free energy estimations, as sampling all relevant configurations would require prohibitively extensive simulations. To enhance the sampling, we decided to perform non‐equilibrium free‐energy calculations, which are repeated many times starting from different initial placements, while averaging the calculated non‐equilibrium values appropriately (Goette & Grubmüller, 2009; Jarzynski, 1997). Bootstrapping analysis has shown that robust estimates of sorption free energy can be obtained from 75 simulations (1.6 ns each, 25 simulations for each independent LHA model) in which the sorbate is grown into the system in a random position, followed by 75 simulations (1.6 ns each, 25 simulations for each independent LHA model), in which it is removed from the system (Figure 3).

Calculated free energies on a set of 18 diverse small molecules (Table 1) show a very good correlation with experimental data for both dry and hydrated Leonardite HA (Figure 4). Various sources of the remaining disagreements can be identified. Primarily, both experimental measurements and computational calculations come with their uncertainties, with the latter estimated at 1.6 and 1.3 kJ mol^−1^ on average for the dry and hydrated LHA systems. Moreover, although the overall chemical composition of the model LHA is in agreement with the real sample, the actual molecular composition probably differs between the two. Note, however, that the same model was used to successfully describe a number of physicochemical properties of Leonardite HA (Petrov et al., 2017). Furthermore, the sorption free energy calculated in the Leonardite system with lower water content (dry LHA) shows equally good level of matching as the hydrated LHA with experiments performed on the hydrated samples (Figure S2). This, together with the excellent agreement between hydration and sorption free energies in the hydrated LHA (Figure S3), suggests that the water content of the hydrated Leonardite HA model might be too high. However, another possible explanation for the observed matching between hydration and sorption free energies would be that, given the low diffusion coefficient of LHA molecules, sorbates colocalize with water patches, which is in agreement with the preferential solvation analysis (Figure 5). If this is the case, the non‐equilibrium simulations of removing a sorbate would potentially be biased, as they predominantly start from a configuration that is not equilibrated. Importantly, taking these potential pitfalls into account, the observed correlation between calculated and experimental sorption free energies is strikingly high. What is more, even though the applied approach suffers from the potential limitations discussed above, it is in principle applicable to any SOM system of interest. Taking this line of argument one step further, modelling sorption processes in even more complex systems can be attempted (e.g., including interfaces between SOM and mineral surfaces). It is worth noting that the Vienna Soil‐Organic‐Matter Modeler (Sündermann et al., 2015), as a versatile tool, can be used to create such models and that mineral surfaces and sorption processes have been simulated using classical force fields already (Samaraweera et al., 2014; Solc, Gerzabek, Lischka, & Tunega, 2011; Teppen et al., 1998).

In addition, using the power of MD simulations to provide microscopic level insight into molecular interactions relevant for the studied processes, we were able to explore how the sorbate molecules associate to nano‐compartments of the Leonardite HA models (Table S1, Figures 6 and 7). This analysis highlighted the significance of distinct interactions of the sorbate with its local environment in the sorption process, in particular the interactions formed between its heteroatoms with water molecules (both polar interactions represented with electrostatic energy and hydrogen bonding) and with heteroatoms of the sorbent. Also, it supports the above‐discussed importance of appropriate localization in non‐equilibrium simulations, as it shows that the surrounding environment strongly affects the sorption propensity. It is tempting to speculate that such information could be utilized to develop an accurate semiempirical prediction model for sorption free energies, where the indicated properties could be used as descriptors.

## 5 CONCLUSIONS

This study provides compelling evidence that molecular dynamics in combination with perturbation free energy methods can be utilized to study sorption processes in SOM systems. Although computationally relatively expensive, it presents a rigorous, physics‐based approach to estimate the sorption free energy, applicable to any SOM system of interest. More importantly, this in‐detail exploration of sorption properties of SOM allows us to provide an atomistic picture behind the relevant processes and shed light on underlying molecular mechanisms. It is our hope that with ever‐growing computer power, such studies in combination with experimental efforts will deepen our understanding of sorption processes and, related to this, the environmental fate of nutrients and pollutants, to ultimately help us achieve better soil management.

## Supporting information


**Figure S1.** Hydration free energy, comparison between calculated and experimental data. Initial parameterization attempt of isopropyl ether and two groups of additional compounds chemically similar to dimethyl succinate and 1‐undecene yielded hydration free energies in discrepancy with the experiment. For that reason, these compounds were reparametrized and the pairs of their calculated hydration free energies (initial parameterization and reparameterization) are connected with vertical dotted lines. Note that the effect of reparameterization of dimethyl succinate bearing two ester groups is much more pronounced (approximately 8 kJ mol^−1^) than is the case for additional similar compounds as they bear one ester group. Compounds (Table 1) are separated into groups based on their chemical composition. The correlation is captured with the black regression line and Pearson coefficient (hydration free energies of initial parameterization not taken into account), while the identity line is shown in green.
**Figure S2.** Sorption free energy in the hydrated Leonardite HA compared to experimental data from dry Leonardite HA and the other way around. Compounds (Table 1) are separated into groups based on their chemical composition. The correlation is captured with the black regression line and Pearson coefficient, while the identity line is shown in green.
**Figure S3.** Comparison between sorption free energy in hydrated Leonardite HA and hydration free energy for calculated (left) and experimental (right) data. Compounds (Table 1) are separated into groups based on their chemical composition. The correlation is captured with the black regression line and Pearson coefficient, while the best fitting line with slope 1 is shown in green. Nota that the same comparison of the hydration to sorption free energy in the dry LHA systems shows weaker agreement, with the correlation coefficients of 0.8 and 0.72 for the calculated and experimental data, respectively (data not shown).
**Figure S4.** Selected microscopic properties calculated from observed interactions in simulated trajectories between sorbate compounds and Leonardite HA and their relation to sorption free energy in the dry Leonardite HA. The correlation between a given property and the sorption free energy for the 18 studied compounds (Table 1) is captured with the black regression line and Pearson coefficient. Similar to Figure 6, properties showing a strong anti‐correlation A‐C, a strong anti‐correlation D and E, and no correlation F–H.
**Table S1.** List of microscopic properties calculated from observed interactions in simulated trajectories between sorbate compounds and the Leonardite HA and their relation to sorption propensity, expressed in terms of the correlation coefficients to the calculated sorption free energy. Analyzed properties include potential energy (*E*) and different components thereof (electrostatic and van der Waals), hydrogen bonds (*H‐bonds*), and Kirkwood‐Buff integrals (*G*) ( marked with a star when non‐normalized) with the limit of 0.3, 0.4 and 0.5 nm between the Leonardite HA and the sorbate and their subparts (carbon and heteroatoms of LHA molecules, water and calcium on one hand and carbon and heteroatoms on the other), as well as solvent‐accessible surface area (*SASA*) of the sorbate with the carbon and heteroatom fractions.

## Data Availability

The data that support the findings of this study are available from the corresponding author upon reasonable request.
