# Supplementary material for: Molecular modelling of sorption processes of a range of diverse small organic molecules in Leonardite humic acid
Source: Eur J Soil Sci. 2019 Aug 20;71(5):831–44. doi: 10.1111/ejss.12868 (PMC7540484; doi:10.1111/ejss.12868)
Supplement: Supplementary file 1 — Figure S1. Hydration free energy, comparison between calculated and experimental data. Initial parameterization attempt of isopropyl ether and two groups of additional compounds chemically similar to dimethyl succinate and 1‐undecene yielded hydration free energies in discrepancy with the experiment. For that reason, these compounds were reparametrized and the pairs of their calculated hydration free energies (initial parameterization and reparameterization) are connected with vertical dotted lines. Note that the effect of reparameterization of dimethyl succinate bearing two ester groups is much more pronounced (approximately 8 kJ mol−1) than is the case for additional similar compounds as they bear one ester group. Compounds (Table 1) are separated into groups based on their chemical composition. The correlation is captured with the black regression line and Pearson coefficient (hydration free energies of initial parameterization not taken into account), while the identity line is shown in green. Figure S2. Sorption free energy in the hydrated Leonardite HA compared to experimental data from dry Leonardite HA and the other way around. Compounds (Table 1) are separated into groups based on their chemical composition. The correlation is captured with the black regression line and Pearson coefficient, while the identity line is shown in green. Figure S3. Comparison between sorption free energy in hydrated Leonardite HA and hydration free energy for calculated (left) and experimental (right) data. Compounds (Table 1) are separated into groups based on their chemical composition. The correlation is captured with the black regression line and Pearson coefficient, while the best fitting line with slope 1 is shown in green. Nota that the same comparison of the hydration to sorption free energy in the dry LHA systems shows weaker agreement, with the correlation coefficients of 0.8 and 0.72 for the calculated and experimental data, respectively (data not shown). Figure S4. S [file EJSS-71-831-s001.docx]

Supporting Information

Molecular modeling of sorption processes of a range of diverse small organic molecules in Leonardite humic acid

Sorption of small organics in Leonardite HA

Drazen Petrov,^1*^ Daniel Tunega,^2,3^ Martin H. Gerzabek,^2^ Chris Oostenbrink^1^

January 2019

^1^Department of Material Sciences and Process Engineering, Institute of Molecular Modeling and Simulation, University of Natural Resources and Life Sciences Vienna, Muthgasse 18, A-1190 Vienna, Austria

^2^Department of Forest- and Soil Sciences, Institute of Soil Research, University of Natural Resources and Life Sciences Vienna, Peter-Jordan-Straße 82, A-1190 Vienna, Austria

^3^School of Pharmaceutical Science and Technology, Tianjin University, Tianjin 300072, People’s Republic of China

*corresponding author

Drazen Petrov

drazen.petrov@boku.ac.at

# SUPPLEMENTARY INFORMATION FIGURES AND TABLES

Figure S1


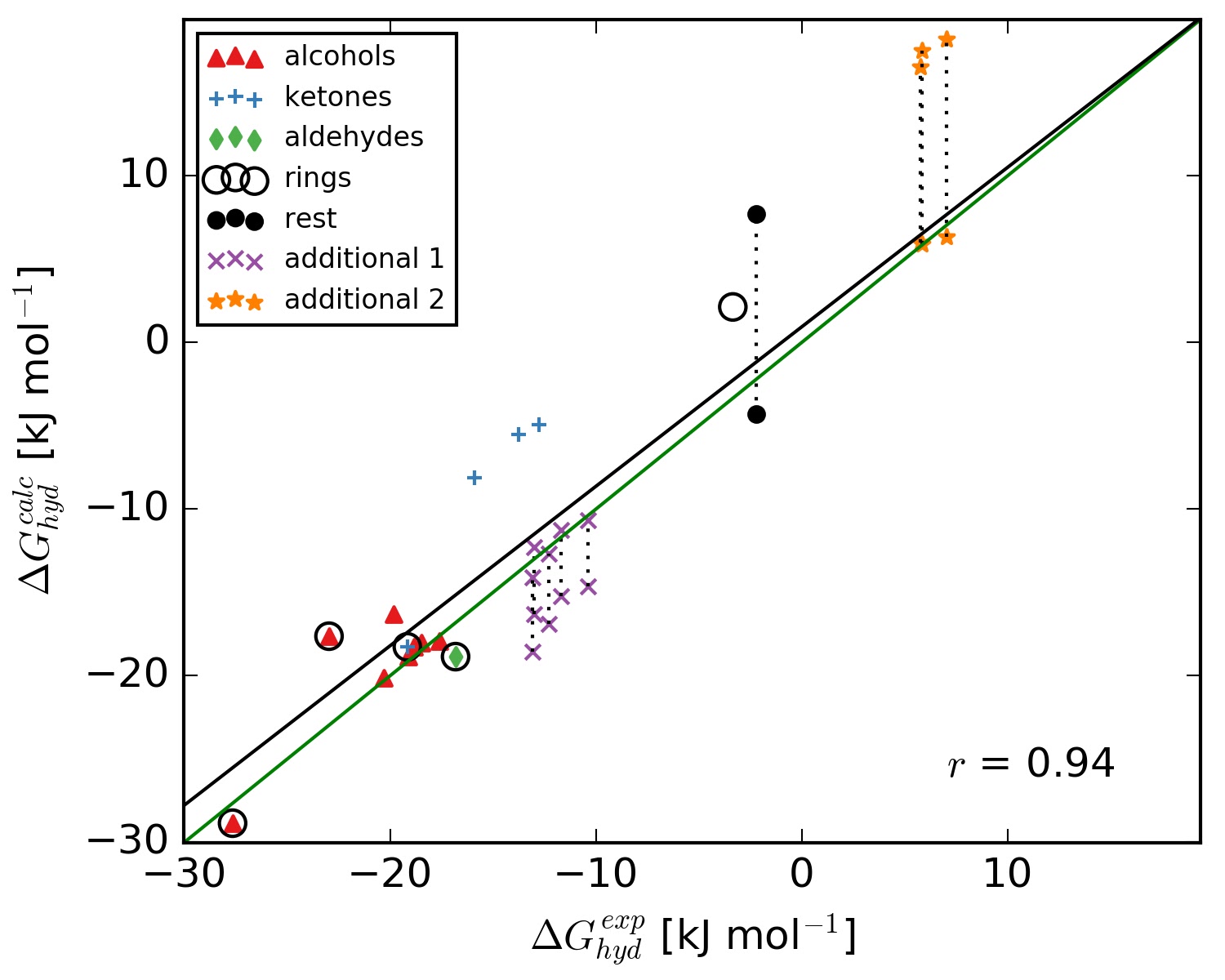


Hydration free energy, comparison between calculated and experimental data. Initial parameterization attempt of isopropyl ether and two groups of additional compounds chemically similar to dimethyl succinate and 1-undecene yielded hydration free energies in discrepancy with experiment. For that reason, these compounds were reparametrized and the pairs of their calculated hydration free energies (initial parameterization and reparameterization) are connected with vertical dotted lines. Note that the effect of reparameterization of dimethyl succinate bearing two ester groups is much more pronounced (approximately 8 kJ mol^-1^) than is the case for additional similar compounds as they bear one ester group. Compounds (Table 1) are separated in groups based on their chemical composition. The correlation is captured with the black regression line and Pearson coefficient (hydration free energies of initial parameterization not taken into account), while the identity line is shown in green.

Figure S2


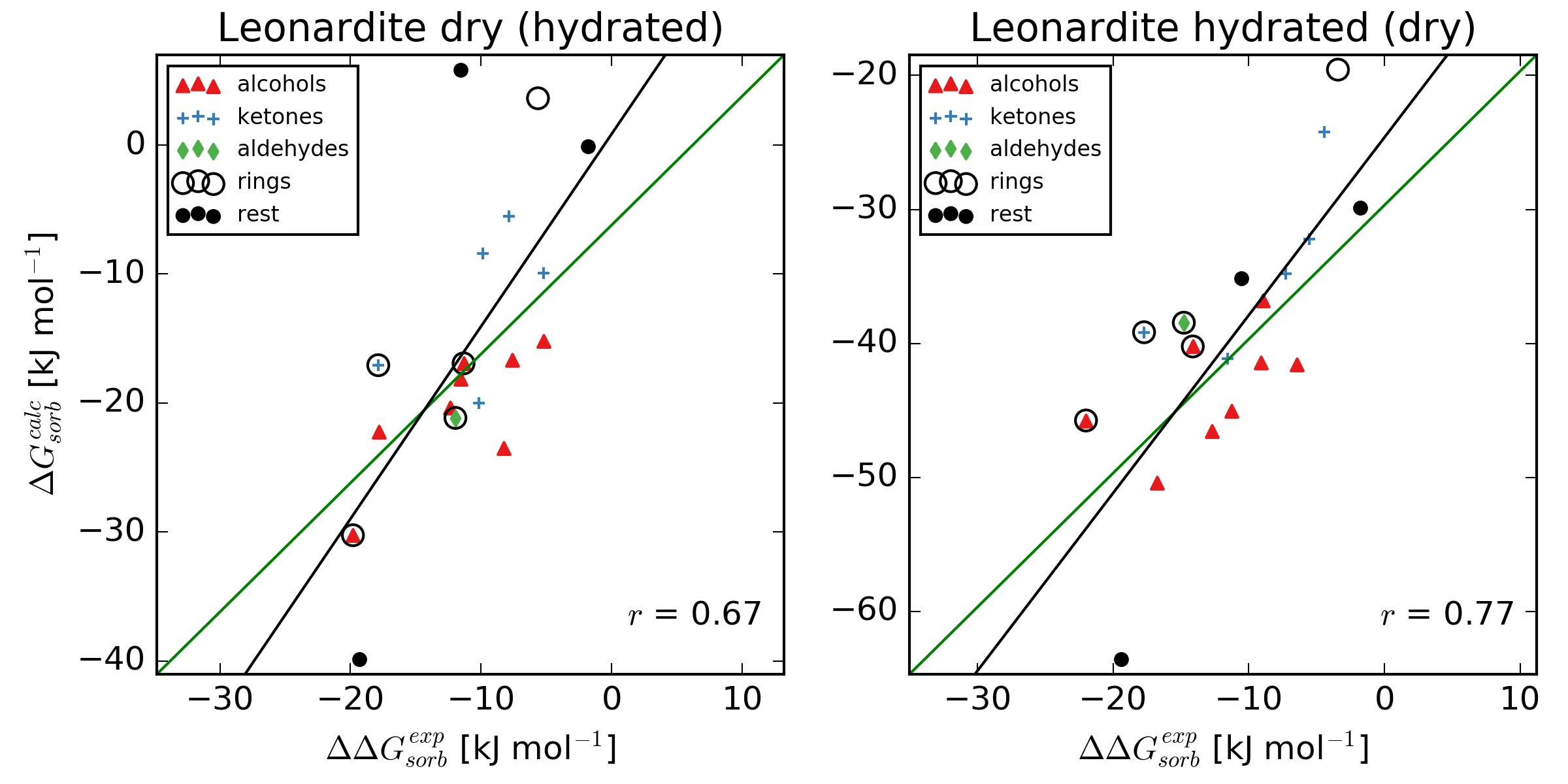


Sorption free energy in the hydrated Leonardite HA compared to experimental data from dry Leonardite HA and the other way around. Compounds (Table 1) are separated in groups based on their chemical composition. The correlation is captured with the black regression line and Pearson coefficient, while the identity line is shown in green.

Figure S3


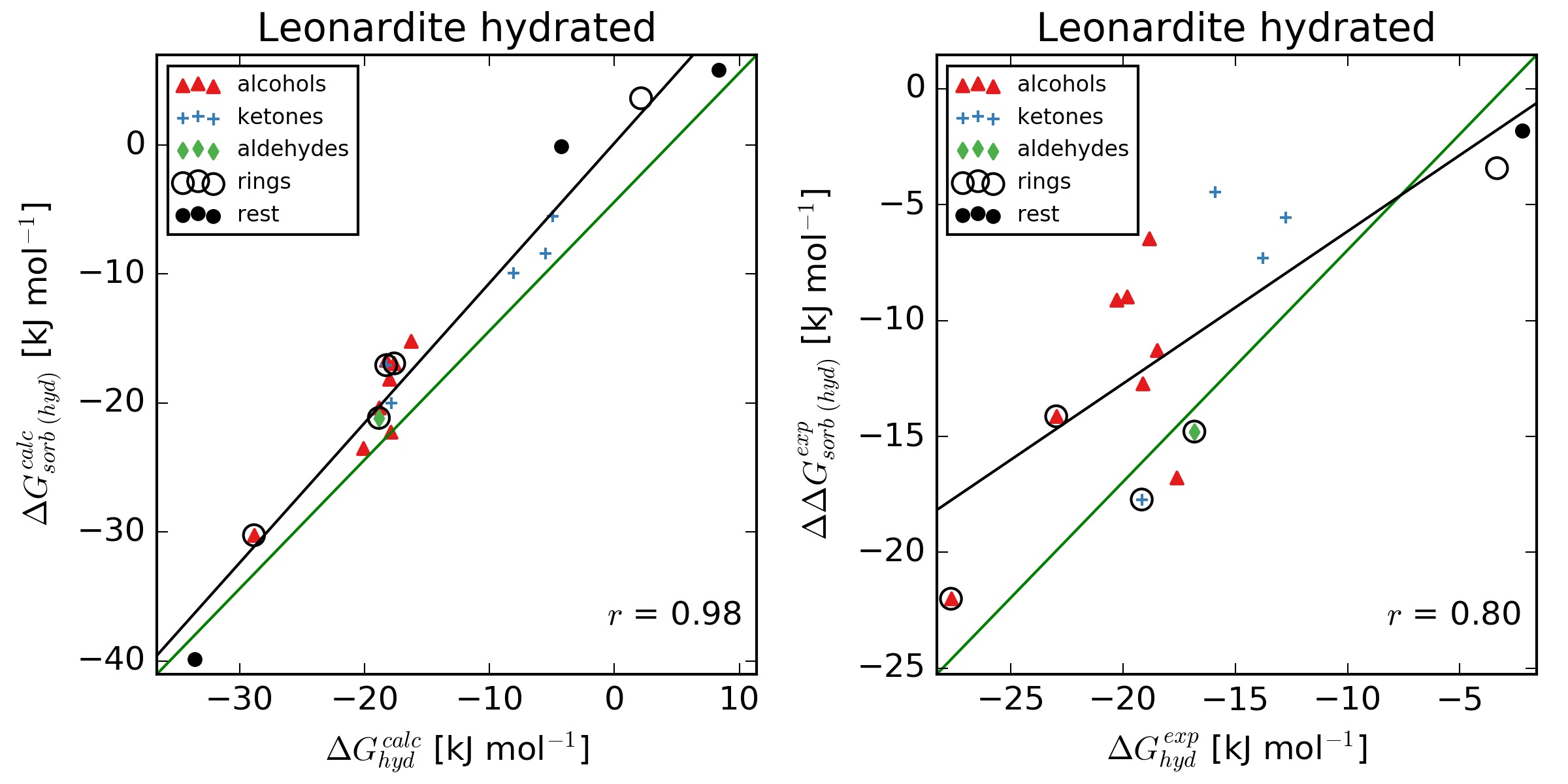


Comparison between sorption free energy in hydrated Leonardite HA and hydration free energy for calculated (left) and experimental (right) data. Compounds (Table 1) are separated in groups based on their chemical composition. The correlation is captured with the black regression line and Pearson coefficient, while the best fitting line with slope 1 is shown in green. Nota that the same comparison of the hydration to sorption free energy in the dry LHA systems show weaker agreement, with the correlation coefficients of 0.8 and 0.72 for the calculated and experimental data, respectively (data now shown).

Figure S4


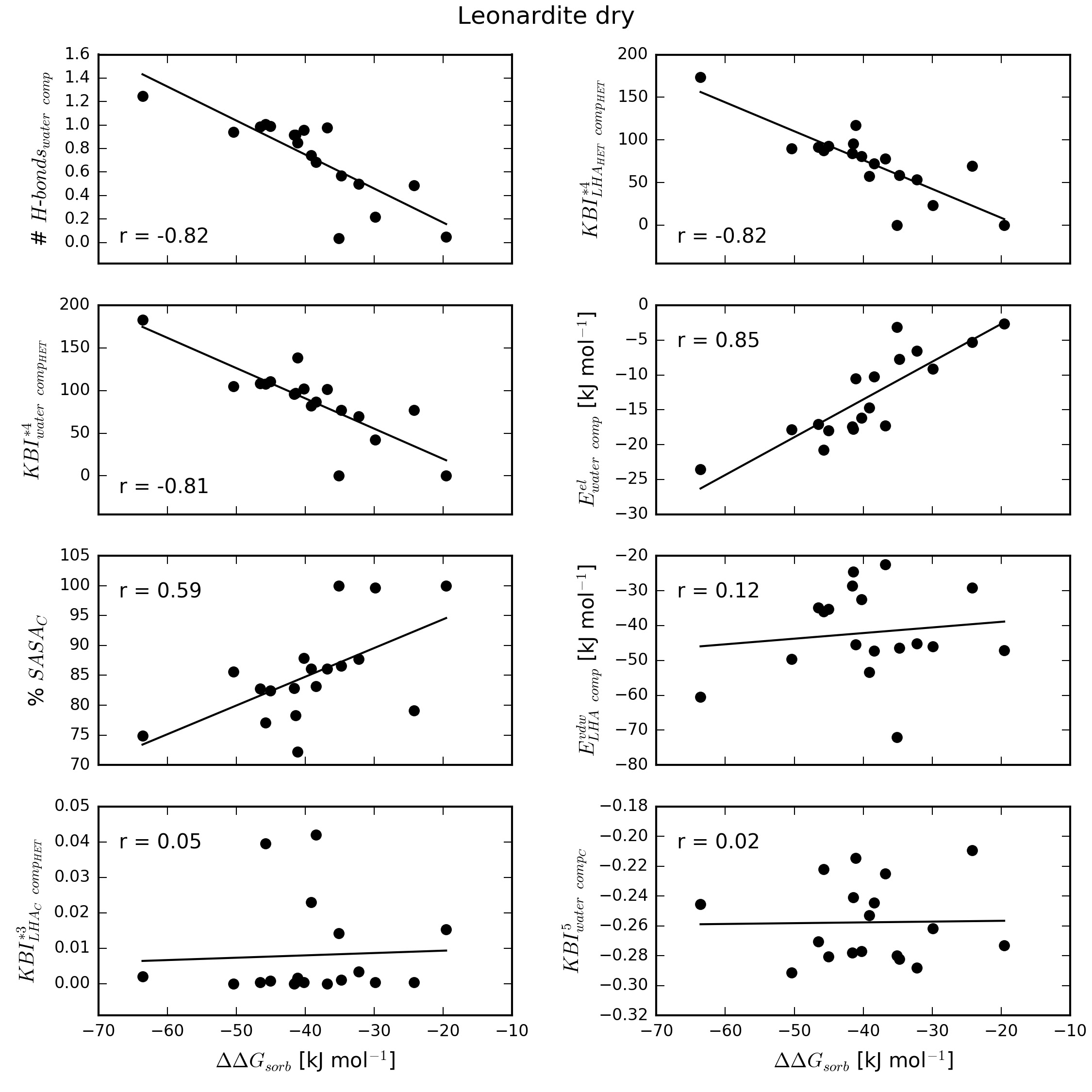


Selected microscopic properties calculated from observed interactions in simulated trajectories between sorbate compounds and Leonardite HA and their relation to sorption free energy in the dry Leonardite HA. The correlation between a given property and the sorption free energy for the 18 studied compounds (Table 1) is captured with the black regression line and Pearson coefficient. Similarly to Figure 6, properties showing a strong anti-correlation A-C, a strong anti-correlation D and E, and no correlation F-H.

Table S1.

| # | property | *r_dry_* | *r_hyd_* | # | property | *r_dry_* | *r_hyd_* |
| --- | --- | --- | --- | --- | --- | --- | --- |
| 1 | $\boldsymbol{E}_{\boldsymbol{sor sor}}^{\boldsymbol{pot}}$ | 0.49 | 0.56 | 34 | $\boldsymbol{G}_{\boldsymbol{LHA}_{\boldsymbol{C}} \boldsymbol{sor}_{\boldsymbol{het}}}^{\boldsymbol{r = 0.4 nm}}$ | -0.44 | -0.64 |
| 2 | $\boldsymbol{E}_{\boldsymbol{sor sor}}^{\boldsymbol{vdW}}$ | 0.24 | -0.23 | 35 | $\boldsymbol{G}_{\boldsymbol{LHA}_{\boldsymbol{C}} \boldsymbol{sor}_{\boldsymbol{het}}}^{\boldsymbol{r = 0.5 nm}}$ | -0.48 | -0.7 |
| 3 | $\boldsymbol{E}_{\boldsymbol{sor sor}}^{\boldsymbol{el}}$ | 0.48 | 0.57 | 36 | $\boldsymbol{G}_{\boldsymbol{LHA}_{\boldsymbol{C}} \boldsymbol{sor}_{\boldsymbol{C}}}^{\boldsymbol{r = 0.3 nm}}$ | 0 | -0.21 |
| 4 | $\boldsymbol{E}_{\boldsymbol{sor LHA}}^{\boldsymbol{pot}}$ | 0.48 | 0.21 | 37 | $\boldsymbol{G}_{\boldsymbol{LHA}_{\boldsymbol{C}} \boldsymbol{sor}_{\boldsymbol{C}}}^{\boldsymbol{r = 0.4 nm}}$ | 0.06 | -0.32 |
| 5 | $\boldsymbol{E}_{\boldsymbol{sor LHA}}^{\boldsymbol{vdW}}$ | 0.12 | -0.22 | 38 | $\boldsymbol{G}_{\boldsymbol{LHA}_{\boldsymbol{C}} \boldsymbol{sor}_{\boldsymbol{C}}}^{\boldsymbol{r = 0.5 nm}}$ | 0.02 | -0.33 |
| 6 | $\boldsymbol{E}_{\boldsymbol{sor LHA}}^{\boldsymbol{el}}$ | 0.46 | 0.44 | 39 | $\boldsymbol{G}_{\boldsymbol{Ca}\boldsymbol{sor}_{\boldsymbol{het}}}^{\boldsymbol{r = 0.3 nm}}$ | 0.14 | -0.19 |
| 7 | $\boldsymbol{E}_{\boldsymbol{sor Ca}}^{\boldsymbol{pot}}$ | 0.1 | 0.47 | 40 | $\boldsymbol{G}_{\boldsymbol{Ca}\boldsymbol{sor}_{\boldsymbol{het}}}^{\boldsymbol{r = 0.4 nm}}$ | -0.04 | -0.22 |
| 8 | $\boldsymbol{E}_{\boldsymbol{sor Ca}}^{\boldsymbol{vdW}}$ | 0.16 | -0.46 | 41 | $\boldsymbol{G}_{\boldsymbol{Ca}\boldsymbol{sor}_{\boldsymbol{het}}}^{\boldsymbol{r = 0.5 nm}}$ | -0.37 | -0.58 |
| 9 | $\boldsymbol{E}_{\boldsymbol{sor Ca}}^{\boldsymbol{el}}$ | 0.09 | 0.48 | 42 | $\boldsymbol{G}_{\boldsymbol{LHA}_{\boldsymbol{het}} \boldsymbol{sor}_{\boldsymbol{C}}}^{\boldsymbol{* r = 0.3 nm}}$ | -0.02 | -0.22 |
| 10 | $\boldsymbol{E}_{\boldsymbol{sor water}}^{\boldsymbol{pot}}$ | 0.93 | 0.38 | 43 | $\boldsymbol{G}_{\boldsymbol{LHA}_{\boldsymbol{het}} \boldsymbol{sor}_{\boldsymbol{C}}}^{\boldsymbol{* r = 0.4 nm}}$ | -0.06 | 0.15 |
| 11 | $\boldsymbol{E}_{\boldsymbol{sor water}}^{\boldsymbol{vdW}}$ | -0.16 | -0.4 | 44 | $\boldsymbol{G}_{\boldsymbol{LHA}_{\boldsymbol{het}} \boldsymbol{sor}_{\boldsymbol{C}}}^{\boldsymbol{* r = 0.5 nm}}$ | 0.04 | 0.32 |
| 12 | $\boldsymbol{E}_{\boldsymbol{sor water}}^{\boldsymbol{el}}$ | 0.85 | 0.86 | 45 | $\boldsymbol{G}_{\boldsymbol{water}\boldsymbol{sor}_{\boldsymbol{het}}}^{\boldsymbol{* r = 0.3 nm}}$ | -0.8 | -0.93 |
| 13 | *# H-bonds_LHA_* | -0.64 | -0.62 | 46 | $\boldsymbol{G}_{\boldsymbol{water}\boldsymbol{sor}_{\boldsymbol{het}}}^{\boldsymbol{* r = 0.4 nm}}$ | -0.81 | -0.86 |
| 14 | *# H-bonds_water_* | -0.82 | -0.95 | 47 | $\boldsymbol{G}_{\boldsymbol{water}\boldsymbol{sor}_{\boldsymbol{het}}}^{\boldsymbol{* r = 0.5 nm}}$ | -0.82 | -0.8 |
| 15 | *SASA* | -0.21 | 0.27 | 48 | $\boldsymbol{G}_{\boldsymbol{LHA}_{\boldsymbol{het}} \boldsymbol{sor}_{\boldsymbol{het}}}^{\boldsymbol{* r = 0.3 nm}}$ | -0.62 | -0.63 |
| 16 | *SASA_C_* | 0.59 | 0.84 | 49 | $\boldsymbol{G}_{\boldsymbol{LHA}_{\boldsymbol{het}} \boldsymbol{sor}_{\boldsymbol{het}}}^{\boldsymbol{* r = 0.4 nm}}$ | -0.82 | -0.88 |
| 17 | *SASA_het_* | -0.59 | -0.84 | 50 | $\boldsymbol{G}_{\boldsymbol{LHA}_{\boldsymbol{het}} \boldsymbol{sor}_{\boldsymbol{het}}}^{\boldsymbol{* r = 0.5 nm}}$ | -0.79 | -0.81 |
| 18 | $\boldsymbol{G}_{\boldsymbol{LHA}_{\boldsymbol{het}} \boldsymbol{sor}_{\boldsymbol{C}}}^{\boldsymbol{r = 0.3 nm}}$ | 0.01 | -0.33 | 51 | $\boldsymbol{G}_{\boldsymbol{Ca}\boldsymbol{sor}_{\boldsymbol{C}}}^{\boldsymbol{* r = 0.3 nm}}$ | -0.23 | 0 |
| 19 | $\boldsymbol{G}_{\boldsymbol{LHA}_{\boldsymbol{het}} \boldsymbol{sor}_{\boldsymbol{C}}}^{\boldsymbol{r = 0.4 nm}}$ | -0.09 | -0.41 | 52 | $\boldsymbol{G}_{\boldsymbol{Ca}\boldsymbol{sor}_{\boldsymbol{C}}}^{\boldsymbol{* r = 0.4 nm}}$ | 0.11 | -0.39 |
| 20 | $\boldsymbol{G}_{\boldsymbol{LHA}_{\boldsymbol{het}} \boldsymbol{sor}_{\boldsymbol{C}}}^{\boldsymbol{r = 0.5 nm}}$ | 0.04 | -0.29 | 53 | $\boldsymbol{G}_{\boldsymbol{Ca}\boldsymbol{sor}_{\boldsymbol{C}}}^{\boldsymbol{* r = 0.5 nm}}$ | 0.2 | 0.22 |
| 21 | $\boldsymbol{G}_{\boldsymbol{water}\boldsymbol{sor}_{\boldsymbol{het}}}^{\boldsymbol{r = 0.3 nm}}$ | -0.42 | -0.59 | 54 | $\boldsymbol{G}_{\boldsymbol{water}\boldsymbol{sor}_{\boldsymbol{C}}}^{\boldsymbol{* r = 0.3 nm}}$ | 0.14 | 0.18 |
| 22 | $\boldsymbol{G}_{\boldsymbol{water}\boldsymbol{sor}_{\boldsymbol{het}}}^{\boldsymbol{r = 0.4 nm}}$ | -0.42 | -0.67 | 55 | $\boldsymbol{G}_{\boldsymbol{water}\boldsymbol{sor}_{\boldsymbol{C}}}^{\boldsymbol{* r = 0.4 nm}}$ | 0.03 | 0.32 |
| 23 | $\boldsymbol{G}_{\boldsymbol{water}\boldsymbol{sor}_{\boldsymbol{het}}}^{\boldsymbol{r = 0.5 nm}}$ | -0.49 | -0.71 | 56 | $\boldsymbol{G}_{\boldsymbol{water}\boldsymbol{sor}_{\boldsymbol{C}}}^{\boldsymbol{* r = 0.5 nm}}$ | 0.04 | 0.39 |
| 24 | $\boldsymbol{G}_{\boldsymbol{LHA}_{\boldsymbol{het}} \boldsymbol{sor}_{\boldsymbol{het}}}^{\boldsymbol{r = 0.3 nm}}$ | -0.43 | -0.49 | 57 | $\boldsymbol{G}_{\boldsymbol{LHA}_{\boldsymbol{C}} \boldsymbol{sor}_{\boldsymbol{het}}}^{\boldsymbol{* r = 0.3 nm}}$ | -0.61 | -0.58 |
| 25 | $\boldsymbol{G}_{\boldsymbol{LHA}_{\boldsymbol{het}} \boldsymbol{sor}_{\boldsymbol{het}}}^{\boldsymbol{r = 0.4 nm}}$ | -0.46 | -0.71 | 58 | $\boldsymbol{G}_{\boldsymbol{LHA}_{\boldsymbol{C}} \boldsymbol{sor}_{\boldsymbol{het}}}^{\boldsymbol{* r = 0.4 nm}}$ | -0.76 | -0.77 |
| 26 | $\boldsymbol{G}_{\boldsymbol{LHA}_{\boldsymbol{het}} \boldsymbol{sor}_{\boldsymbol{het}}}^{\boldsymbol{r = 0.5 nm}}$ | -0.49 | -0.73 | 59 | $\boldsymbol{G}_{\boldsymbol{LHA}_{\boldsymbol{C}} \boldsymbol{sor}_{\boldsymbol{het}}}^{\boldsymbol{* r = 0.5 nm}}$ | -0.76 | -0.77 |
| 27 | $\boldsymbol{G}_{\boldsymbol{Ca}\boldsymbol{sor}_{\boldsymbol{C}}}^{\boldsymbol{r = 0.3 nm}}$ | -0.23 | 0 | 60 | $\boldsymbol{G}_{\boldsymbol{LHA}_{\boldsymbol{C}} \boldsymbol{sor}_{\boldsymbol{C}}}^{\boldsymbol{* r = 0.3 nm}}$ | 0.05 | -0.14 |
| 28 | $\boldsymbol{G}_{\boldsymbol{Ca}\boldsymbol{sor}_{\boldsymbol{C}}}^{\boldsymbol{r = 0.4 nm}}$ | 0.21 | -0.37 | 61 | $\boldsymbol{G}_{\boldsymbol{LHA}_{\boldsymbol{C}} \boldsymbol{sor}_{\boldsymbol{C}}}^{\boldsymbol{* r = 0.4 nm}}$ | 0.05 | 0.09 |
| 29 | $\boldsymbol{G}_{\boldsymbol{Ca}\boldsymbol{sor}_{\boldsymbol{C}}}^{\boldsymbol{r = 0.5 nm}}$ | 0.26 | -0.16 | 62 | $\boldsymbol{G}_{\boldsymbol{LHA}_{\boldsymbol{C}} \boldsymbol{sor}_{\boldsymbol{C}}}^{\boldsymbol{* r = 0.5 nm}}$ | 0.04 | 0.26 |
| 30 | $\boldsymbol{G}_{\boldsymbol{water}\boldsymbol{sor}_{\boldsymbol{C}}}^{\boldsymbol{r = 0.3 nm}}$ | 0.09 | 0.03 | 63 | $\boldsymbol{G}_{\boldsymbol{Ca}\boldsymbol{sor}_{\boldsymbol{het}}}^{\boldsymbol{* r = 0.3 nm}}$ | 0.05 | -0.43 |
| 31 | $\boldsymbol{G}_{\boldsymbol{water}\boldsymbol{sor}_{\boldsymbol{C}}}^{\boldsymbol{r = 0.4 nm}}$ | 0.02 | -0.26 | 64 | $\boldsymbol{G}_{\boldsymbol{Ca}\boldsymbol{sor}_{\boldsymbol{het}}}^{\boldsymbol{* r = 0.4 nm}}$ | -0.14 | -0.5 |
| 32 | $\boldsymbol{G}_{\boldsymbol{water}\boldsymbol{sor}_{\boldsymbol{C}}}^{\boldsymbol{r = 0.5 nm}}$ | 0.02 | -0.14 | 65 | $\boldsymbol{G}_{\boldsymbol{Ca}\boldsymbol{sor}_{\boldsymbol{het}}}^{\boldsymbol{* r = 0.5 nm}}$ | -0.68 | -0.89 |
| 33 | $\boldsymbol{G}_{\boldsymbol{LHA}_{\boldsymbol{C}} \boldsymbol{sor}_{\boldsymbol{het}}}^{\boldsymbol{r = 0.3 nm}}$ | -0.32 | -0.34 |  |  |  |  |

List of microscopic properties calculated from observed interactions in simulated trajectories between sorbate compounds and the Leonardite HA and their relation to sorption propensity, expressed in terms of the correlation coefficients to the calculated sorption free energy. Analyzed properties include potential energy (*E*) and different components thereof (electrostatic and van der Waals), hydrogen bonds (*H-bonds*), and Kirkwood-Buff integrals (*G*) ( marked with a star when non-normalized) with the limit of 0.3, 0.4 and 0.5 nm between the Leonardite HA and the sorbate and their subparts (carbon and heteroatoms of LHA molecules, water and calcium on one hand and carbon and heteroatoms on the other), as well as solvent-accessible surface area (*SASA*) of the sorbate with the carbon and heteroatom fractions.
